# Starch can expedite the screening for bacterial aflatoxin degraders

**DOI:** 10.1038/s41598-024-83511-3

**Published:** 2024-12-30

**Authors:** Natalie Sandlin, Babak Momeni

**Affiliations:** Biology Department, Boston College, 140 Commonwealth Ave, Chestnut Hill, MA 02467 USA

**Keywords:** Applied microbiology, Food microbiology

## Abstract

Aflatoxins (AFs) are secondary fungal metabolites that contaminate common food crops and are harmful to humans and animals. The ability to degrade or remove aflatoxins from common feed commodities will improve health standards and counter the economic drain inflicted by AF contamination. Bioremediation is a promising solution to AF contamination because of its low cost and few undesired environmental side-effects. Identifying new degrader species is highly beneficial in that it can offer alternatives to overcome the limitations of existing biodegraders, such as narrow working conditions and low degradation rates. Here, we screen several environmental isolates for their AF detoxification ability, using aflatoxin G_2_. We use different carbon sources (glucose and starch) in isolation and culturing media to examine the effect of the environment on degradation ability. Strains isolated in media with starch as the primary carbon source showed a higher percentage of good AF degraders, 16% compared to 2% when glucose was the primary carbon source. Additionally, the majority of species isolated in glucose medium exhibited improved degradation efficiency when moved into starch medium, with one isolate improving degradation levels from 30 to 70%. Our starch screen also revealed three previously unidentified AF degrader bacterial species. Good aflatoxin G_2_ degraders also appear to perform well against aflatoxin B_1_. Overall, for AF degradation, starch medium expedites the screening process and generally improves the performance of isolates. We thus propose that using starch as the carbon source is a promising means to identify new AF degraders in the environment.

**Supplementary Information:**

The online version contains supplementary material available at 10.1038/s41598-024-83511-3.

## Introduction

Aflatoxins (AFs) are secondary fungal metabolites produces by *Aspergillus* spp. that ubiquitously contaminate common food and feed crops^1,2^. Consumption of contaminated foods by animals and humans are detrimental to health since they cause immunodepression, cancers, and hormone imbalances^3^. Current physical and chemical methods for decontamination suffer from high costs, low reproducibility and efficiency, and the potential to diminish nutrients in the food^4–9^. A more promising solution for AF decontamination is through biological methods, e.g. bioremediation using microbes^10–13^. Bioremediation has the potential to offer lower cost, increased efficiency, and safer application in agriculture.

A number of bacterial and fungal species have been identified as AF degraders^12,14–16^, yet none have been effectively implemented for commercial use. The issues currently faced in using these species is a limited knowledge of their working conditions and degradation mechanisms^17,18^. Previous reports have identified two general categories of removal of AFs^19^: through adsorption to microbial cell components or enzymatic degradation. Adsorption has been observed in Lactobacillaceae and Bifidobacteriaceae bacterial families and in Pichiaceae and Saccharomycetaceae fungal families^19^. Instances of identified enzymatic AF degradation have been more phylogenetically diverse, with organisms from Bacillaceae, Pseudomonadaceae, Lactobacillaceae, Staphylococcaceae, Enterobacteriaceae, Corynebacteriaceae bacterial families and Polyporaceae, Pichiaceae, and Aspergillaceae fungal families^19^. Enzymes responsible for AF degradation often belong to the broad category of oxidoreductases (including laccases, oxidases, reductases, and peroxidases) and lactonases^20^. Other enzymes outside of these two categories have also been found, such as PADE_1_ isolated from *Pantoea*^21^ and PADE_2_ isolated from *Pseudomonas*^22^. However, the phylogenetic diversity and enzymatic diversity of AF degradation is still under investigation. It is highly beneficial to identify new degraders and their mechanisms of degradation to expand the existing knowledge and potentially find species with higher AF degradation performance.

The process of finding new degrader species has previously been achieved through screening environmental isolates either on the target toxin itself or compounds with a similar structure, such as coumarin in place of AF^14^. However, such screens can be costly and result in limited numbers of degraders identified.

Here, we propose that the ability of a microbe to grow on starch as a more complex carbon source (compared to glucose) can indicate its potential to degrade aflatoxins. The use of a more complex carbon source is motivated by the speculation that enzymes involved in breaking down complex carbon structures are likely to also degrade the hard-to-degrade structure of aflatoxins. In the past, enzymes such as laccase from *Trametes versicolor*, which its primary function is to degrade complex plant carbon sources, have been found to also degrade aflatoxin^23–25^, underscoring this opportunity. Starch in particular is a good choice as a carbon source, because it is safe to handle, cost-effective, and water-soluble, allowing rapid and high throughput screening of isolates. Previous work in our lab has shown that simply changing microbes from a medium with glucose to a medium with starch as its sole carbon source increased degradation performance by strains^26^. The presence of starch in the medium, compared to other carbon sources such as glucose, has also been shown to improve the AF degradation performance by fungus *Aspergillus niger*^27^ and bacterium *Myroides odoratimimus*^28^. Building on this background, we first screened environmental bacterial isolates from several sources for their ability to grow on starch defined medium and then tested for aflatoxin degradation by those able to grow in this medium. In parallel, we screened isolates on glucose defined medium prior to testing for degradation to determine differences in the strains obtained from different carbon sources. We used the native fluorescence of aflatoxins to quantitatively characterize aflatoxin degradation by isolates. This degradation assay showed that a higher percentage of strains isolated on starch had degradation capability compared to those isolated in a medium with glucose as the main carbon source. Additionally, the degradation performance of glucose isolates improved when tested in a starch environment in the degradation assay. These results indicate that starch can be utilized as a cost-effective screening tool for aflatoxin degraders and that environmental conditions such as carbon source can significantly impact degradation rates.

## Results

### Selection on starch identified a greater percentage of good aflatoxin degraders

**Fig. 1:**
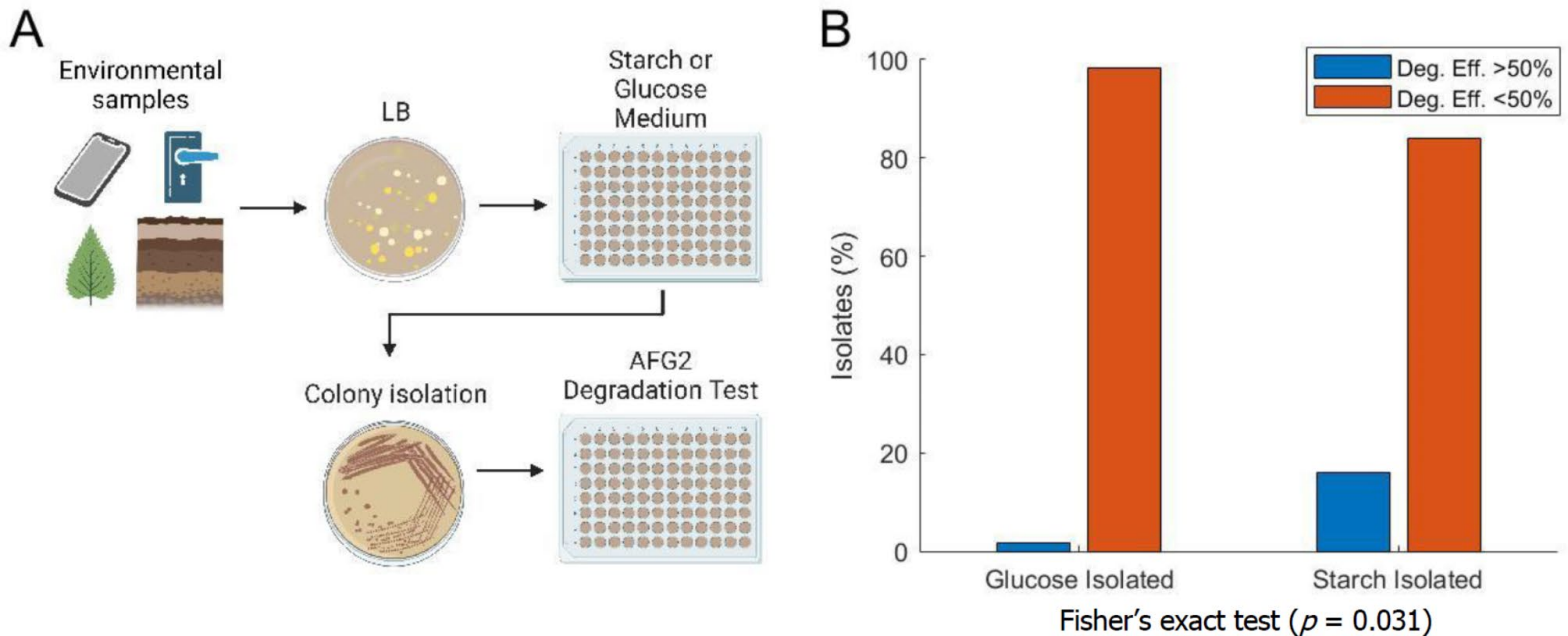
Profile of isolates from starch and glucose screens exhibits superior AFG_2_ degradation performance for those selected in media containing starch as the main carbon source. (**A**) Experimental design schematic showing workflow from sample collection to isolate testing for AF degradation (Created with BioRender.com). (**B**) Breakdown of degradation performance of environmental isolates based on selection medium. Blue bars show isolates that were able to degrade greater than 50% AF in the degradation assay and orange bars show isolates with less than 50% AF degradation. *p* = 0.031, Fisher’s exact test, comparing the number of good degraders arising from total isolates able to grow in glucose versus starch medium.

Environmental samples were taken from various locations in the surrounding area of Chestnut Hill, MA to broadly screen different species and environments for natural AF degraders: soil, leaf, sidewalk, doorknobs, and phone screens (see Methods). After initial culturing on a rich solid (agar) medium, individual colonies (distinguished by different colony morphologies) were inoculated in a defined medium containing either starch or glucose as the sole carbon source (Fig. 1A). Out of 50 isolates tested for growth in starch, 26 were culturable and we subsequently tested them for aflatoxin degradation via our fluorescent AF degradation assay (Table 1). We chose to first screen for AFG_2_ degradation, because compared to AFB_1_, AFG_2_ has stronger fluorescence and lower toxicity, making it a suitable option for high throughput screening in the laboratory. We show later (Fig. 4) that the degradation of AFG_2_ is correlated with the degradation of AFB_1_, further justifying this choice.

Only one starch isolate was unable to degrade AFG_2_ after 72 h of testing and the 24 of the other 25 isolates showed degradation, with 4 having degradation efficiency > 50% (Fig. 1B). Among glucose isolates, a larger percentage of tested isolates grew in the glucose medium (56 of 67), however, eight were unable to degrade AFG_2_ and only 1 showed degradation > 50% (Table 1; Fig. 1B), indicating that fewer active degraders arise in the glucose screen. The odds of finding good degraders in the starch screen is significantly higher than the glucose screen, based on Fisher’s exact test, *p* = 0.031 (Fig. 1B).

**Table 1:** Starch and glucose screen results.

Selection medium	# of isolates	Grow in defined medium	Degradation performance		
			None	Poor (< 50%)	Good (> 50%)
Starch	50	25	1	20	4
Glucose	67	55	8	46	1

### Newly identified aflatoxin degrading species arise from the starch screen

After testing for AF degradation by isolates in their isolation medium, 15 isolates from each screen were semi-randomly selected for further analysis to understand the general trends of the species that arose from each screen. Selected strains were chosen to represent the spectrum of degradation profiles, with representatives of the best, worst, and average performers. These selected isolates had their DNA extracted and PCR amplified for 16S rRNA gene sequencing to determine strain identity (Table 2).

**Table 2:** Isolate identification and degradation profile. Isolates were identified through 16S rRNA gene sequencing. Identification shown in the table are closest match through BLASTn. Species designation is shown in brackets to emphasize that definite species identification is not possible from 16S gene sequencing alone. The order of strains in the table is based on their AF degradation performance in starch medium.

	Isolate	16S rRNA identification	Percent identity	Isolated from	Live degradation (%)	
					In starch	In glucose
Starch isolates	SI-C4	*Stenotrophomonas [maltophilia]*	84.43	Soil	64	13
	SI-B3	*Stenotrophomonas [cyclobalanopsidis]*	96.99	Soil	63	28
	SI-C3	*Citrobacter [cronae]*	79.27	Soil	59	11
	SI-B2	*Stenotrophomonas [lactitubi]*	85.43	Soil	53	39
	SI-E10	*Acinetobacter [oleivorans]*	97.43	Soil	31	13
	SI-C2	*Enterobacter [asburiae]*	97.43	Soil	29	27
	SI-C5	*Pseudomonas [fulva]*	98.77	Soil	22	29
	SI-D4	*Pseudomonas [faucium]*	97.79	Tree trunk	20	8.1
	SI-B10	*Klebsiella [aerogenes]*	97.74	Soil	20	17
	SI-B9	*Klebsiella [aerogenes]*	96.97	Soil	20	17
	SI-G9	*Acinetobacter [geminorum]*	98.16	Sidewalk	18	13
	SI-D6	*Pseudoxanthomonas[ putridarboris]*	96.67	Doorknob	16	31
	SI-C8	*Pseudomonas [urethralis]*	96.55	Soil	11	11
	SI-B4	*Stenotrophomonas [pavanii]*	97.05	Soil	10	25
	SI-C6	*Comamonas [sediminis]*	94.08	Soil	3.8	19
Glucose isolates	GI-38	*Rhodococcus [erythropolis]*	95.81	Soil	84	82
	GI-55	*Bacillus [xiamenensis]*	95.79	Leaf	72	30
	GI-1	*Pseudomonas [oryzihabitans]*	99.69	Phone screen	47	34
	GI-5	*Staphylococcus [epidermidis]*	99.31	Phone screen	40	0.0
	GI-6	*Priestia [flexa]*	97.84	Doorknob	39	35
	GI-9	*Pseudomonas [baltica]*	95.39	Leaf	39	33
	GI-56	*Bacillus [altitudinis]*	99.13	Soil	38	35
	GI-50	*Pseudomonas [umsongensis]*	97.32	Snow	35	32
	GI-33	*Bacillus [sanguinis]*	96.12	Soil	34	32
	GI-37	*Pantoea [agglomerans]*	94.60	Soil	33	33
	GI-17	*Bacillus [aerius]*	94.93	Leaf	33	32
	GI-14	*Pseudomonas [glycinis]*	99.19	Leaf	32	28
	GI-25	*Pantoea [cypripedii]*	94.70	Snow	31	28
	GI-16	*Bacillus [clarus]*	92.02	Leaf	28	11
	GI-51	*Pseudomonas [mandelii]*	84.52	Snow	19	29

Of the glucose isolates analyzed, species of *Pseudomonas*, *Staphylococcus*, *Bacillus*, and *Pantoea* (as determined by 16S rRNA gene sequencing) were present, all genera having previously been identified as aflatoxin degraders^21,22,29–34^. Of starch isolates, while previously identified aflatoxin degrader genera were present, we also found species of *Citrobacter* and *Acinetobacter* which have not been previously implicated as aflatoxin degraders. To our knowledge, this is the first report of species in these two genera to possess aflatoxin degradation ability, although species from these two genera have been shown to degrade other mycotoxins: an *Acinetobacter* sp. isolated from soil degraded zearalenone^35^, and a *Citrobacter* sp. isolated from soil degraded deoxynivalenol^36^. Some of the identified isolates match previous literature at the genus level, and based on 16S rRNA gene sequencing we are not able to confirm or rule out if these isolates match the previously identified AF-degrading species. Overall, the starch screen resulted in newly identified degrader species while the glucose screen did not.

**Fig. 2:**
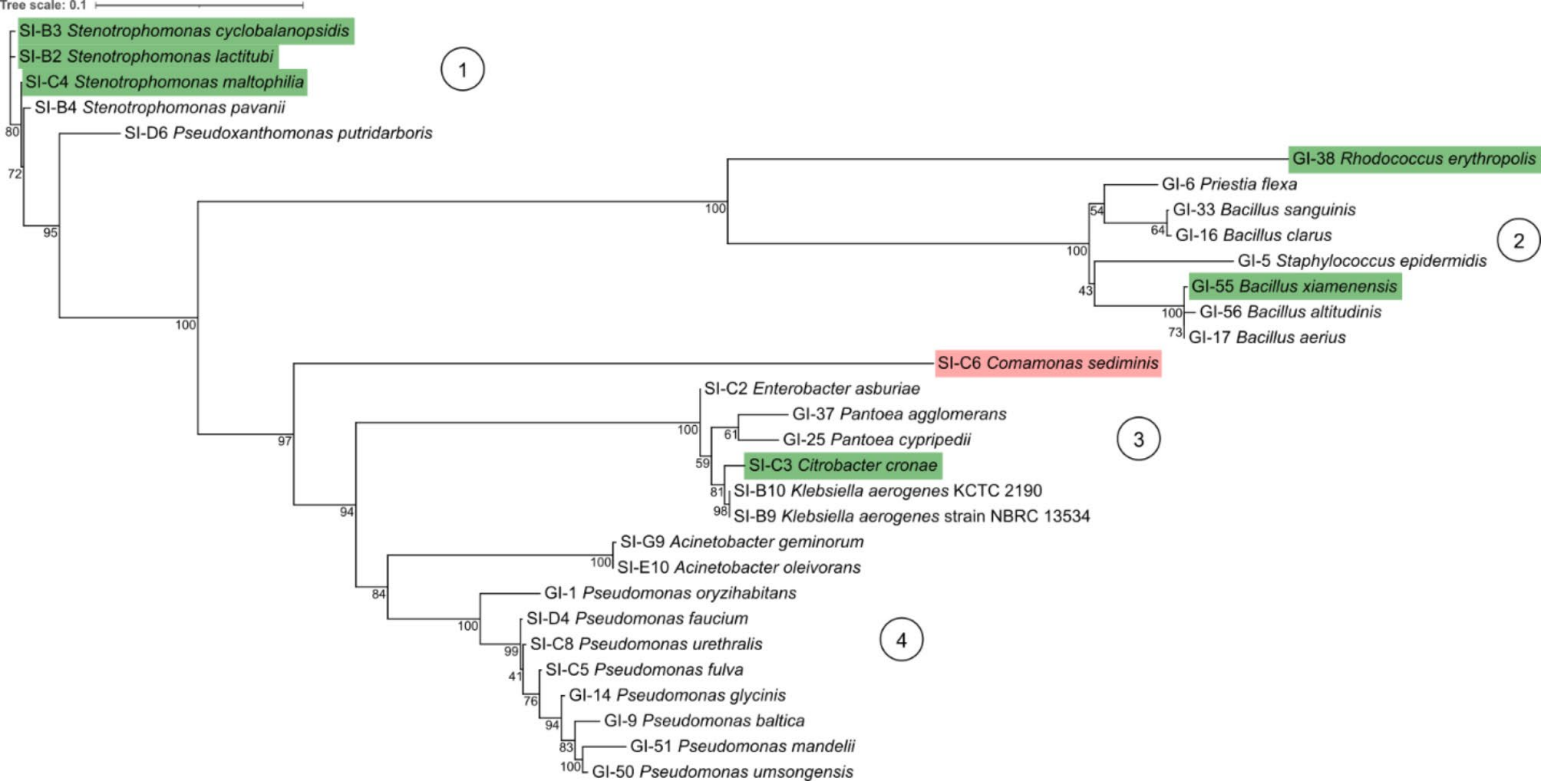
Good degraders are not phylogenetically distinct from poor degraders. Phylogenetic tree of best matched 16S rRNA gene sequences from GenBank database corresponding to glucose and starch isolates is shown. Green highlighted isolates are considered good degraders in starch medium, while red highlighted isolates are non-degraders in starch medium. GI and SI prefixes indicate those isolated in glucose and starch media, respectively. The sequence corresponding to SI-B2 was arbitrarily used as the root. For each branch node, the confidence value based on a bootstrap algorithm is shown in percentages (*n*_*bootstrap*_= 1000). For our strains, there are primarily four clusters of closely related species: (1) Xanthomonadaceae family, (2) Bacillales and Caryophanales orders, (3) Enterobacterales order, and (4) Pseudomonadales order, with isolates GI-38 and SI-C6 outside of these clusters.

We examined the phylogenetic distribution of the AF-degrading taxa that we found in our screens. Interestingly, the majority of isolates we found (21 out of 30) belonged to the phylum Pseudomonadota. The second prevalent taxonomic category was the phylum Firmicutes, due to the number of *Bacillus* species that were identified (5 out of 30). Overall, the distribution of isolates across many phyla indicates a broad ability of bacteria to degrade aflatoxin, without specific taxa-based indications for this ability. Notably, species that showed the strongest AF degradation performance did not group together and were dispersed throughout the phylogenetic tree (Fig. 2, green). However, the one isolate that showed no degradation in starch was taxonomically distant from other isolates (Fig. 2, red). Further examination of the phylogenetic tree in Fig. 2 shows strains primarily in four clusters (Fig. 2): (1) Xanthomondaceaea family (all from the starch screen, multiple good degraders), (2) Bacillales and Caryophanales orders (all from the glucose screen, occasional good degraders), (3) Enterobacterales order (mix of isolates from the starch and glucose screens, occasional good degraders), and (4) Pseudomonadales order (mix of isolates from the starch and glucose screens, with no observed good degraders). Isolates GI-38 and SI-6 were phylogenetically distinct from these clusters, showing good and poor degradation performance, respectively.

### Starch medium, compared to glucose, improves the degradation performance of isolates

**Fig. 3:**
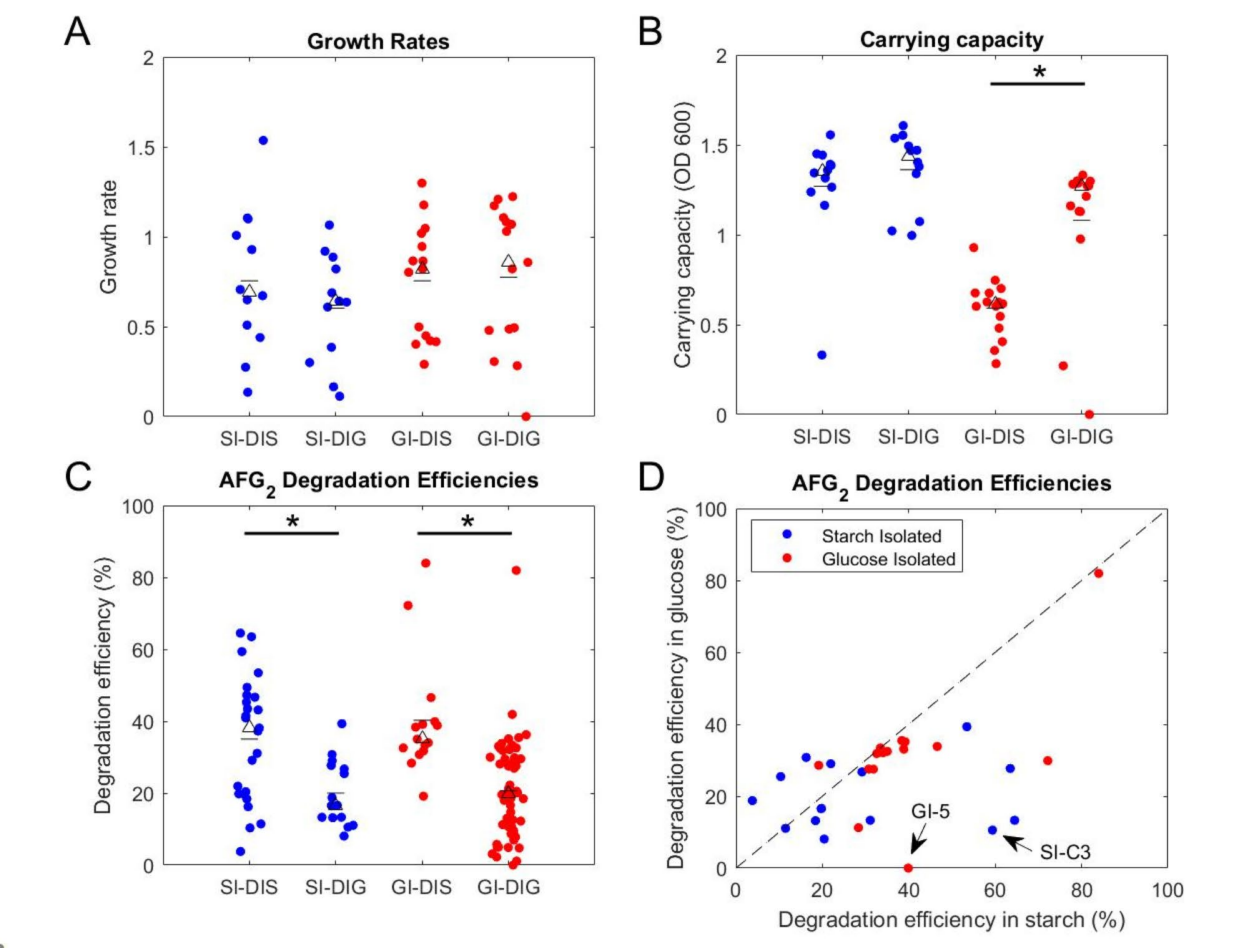
AFG_2_ degradation is improved for glucose-isolated strain when tested in starch medium. Isolates were tested for their growth and AF degradation efficiency when grown in starch and glucose defined media. Starch isolated strains (SI) are shown in blue and glucose isolated strains (GI) are shown in red. Testing in starch medium is indicated by DIS and testing in glucose medium is indicated by DIG. (**A**) Growth rates. (**B**) Carrying capacity. (**C**) Degradation efficiency, shown as percent AF degraded in 48 h. In (**A**–**C**), the marked triangles indicate the group’s median, while the marked dash indicates the group’s mean. (**D**) Degradation efficiencies for each isolate in both media. The dotted line represents the same efficiency between the two media. Each dot is the mean of 2 replicates per culturing condition. *p<0.05, Mann-Whitney U test.

To understand how the environmental carbon source influences degradation, isolates that had undergone 16S rRNA gene sequencing were tested for aflatoxin degradation performance in the opposite medium from their isolation. Starch isolates had significantly lower degradation efficiency when tested in glucose medium (Fig. 3C, blue), and glucose isolates had significantly increased degradation efficiency when tested in starch (Fig. 3C, red). We further examined how the degradation performance of individual strains changed when tested in starch versus glucose media (Fig. S2). We found that even at the level of individual isolates, a majority of isolates show better degradation in the starch medium, compared to the glucose medium (Fig. S2).

Additionally, when looking at growth characteristics for isolates between the two medium types, growth rates remained similar for both glucose- and starch-isolates (Fig. 3A). While carrying capacity remained the same for starch isolates in both media, glucose isolates had significantly lower carrying capacity in starch compared to glucose (Fig. 3B). When examined at the level of individual strains (Fig. S3), we found that growth rates were not clearly likely to become higher or lower in starch versus glucose media. Starch isolates were also not clearly likely to reach a higher or lower carrying capacity at the level of individual isolates. However, glucose isolates were more likely to have a higher carrying capacity in glucose medium, compared to starch medium (Fig. S3).

Taken altogether, these dynamics of growth and degradation indicate that lower cell density is not the cause of decreased degradation for the starch isolates in glucose medium, and that for glucose isolates, a lower cell density in starch out-perform the higher cell density of glucose culturing. This increase in performance is likely the result of a metabolism shift when moved to a more complex carbon environment rather than impact on growth since growth rates and carrying capacity remained similar.

Looking closer at individual isolates in glucose and starch media, we see the effect that testing in a starch medium has on degradation capacity. As an example, isolate GI-5 showed no degradation when tested in glucose but showed about 40% degradation in starch (Fig. 3D, highlighted). Additionally, isolate SI-C3, a newly identified aflatoxin degrader, decreased its degradation from 60 to 28% when moved into glucose (Fig. 3D, highlighted), which indicates that in a screen using glucose, this new degrader would likely not have been identified. Overall, the fraction of strains that showed higher degradation in starch versus glucose was 73% (11 out of 15) among starch isolates and 93% (14 out of 15) among glucose isolates (Fig. 3D).

### Isolates show detoxification ability on other aflatoxins

**Fig. 4:**
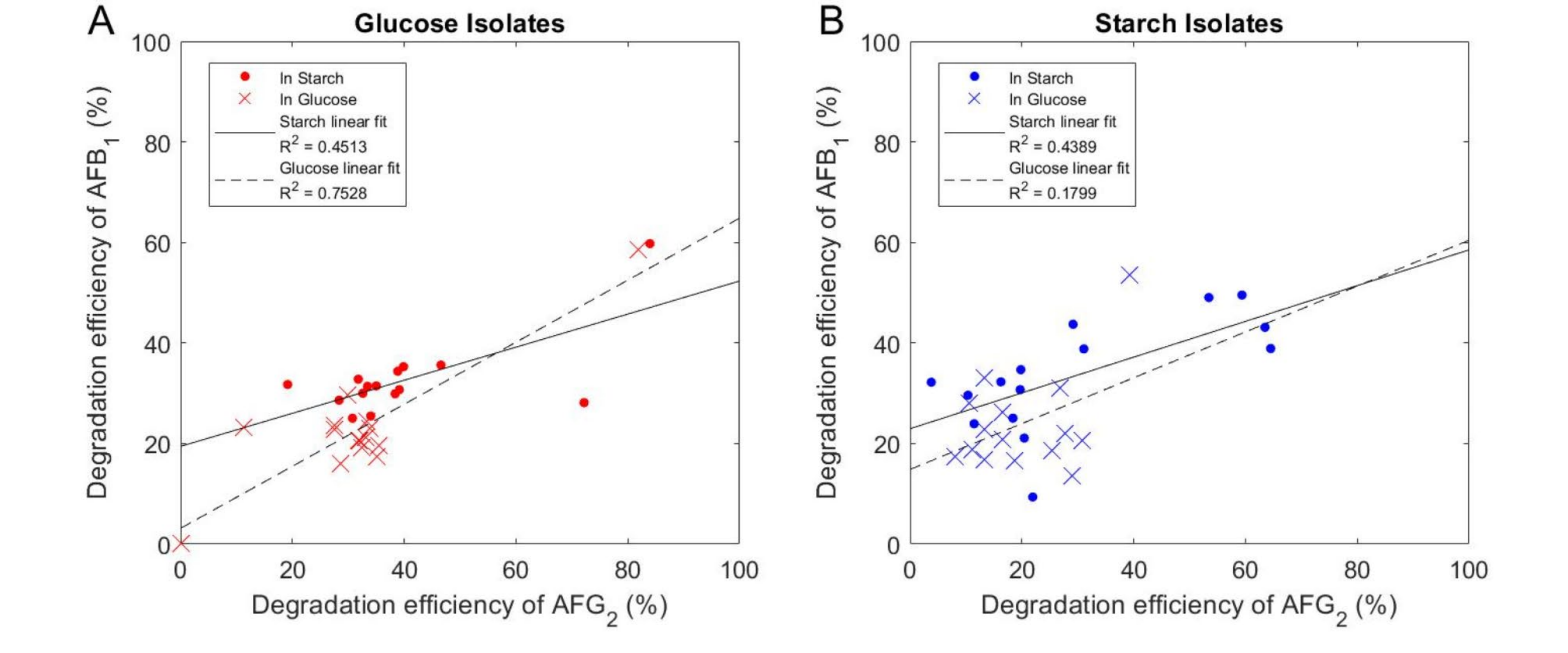
Good AFB_1_ degraders often show good AFG_2_ degradation efficiency. Isolates were tested for their degradation efficiency on two types of aflatoxin, AFB_1_ and AFG_2_, when grown in starch and glucose defined media. Degradation efficiency is shown as percent AF degraded in 48 h for (**A**) glucose isolated strains and (**B**) starch isolated strains. Dots (⋅) represent testing in starch medium and crosses (×) represent testing in glucose medium. Each point is the mean of 2 replicates per culturing condition. Data points on the top-right portion of the plots designate isolates with good performance for AFB_1_ and AFG_2_ degradation.

One key function of a good aflatoxin degrader is the ability to degrade different aflatoxin types. Previous data focused on AFG_2_ since its stronger fluorescence is more reliably detected in our degradation assay. Here, we tested both starch and glucose isolates for their AFB_1_ degradation efficiency to understand the relationship between degradation of these two aflatoxin types. For both sets of isolates in glucose and starch media, even though the efficiency of AFG_2_ degradation is often higher, we generally find that the best degraders of AFB_1_ are the same as the best AFG_2_ degraders (Fig. 4).

In Fig. 4, these good degraders are represented by data points on the top-right portion of the plot. Since the best performing isolates for AFG_2_ degradation typically performed well for AFB_1_ degradation, this justifies the use of AFG_2_ in the screen as a proxy for AF degradation (Fig. 4). The correlation between AFG_2_ and AFB_1_ degradation is less apparent in the glucose medium for starch isolates, with R^2^ = 0.1799 (Fig. 4B). For glucose isolates tested in glucose medium, the relationship between AFG_2_ and AFB_1_ degradation is much stronger, R^2^ = 0.7528 (Fig. 4A). Additionally, when comparing the overall performance of isolates on AFB_1_ in starch and glucose media, a similar trend to AFG_2_ is seen in that testing in starch significantly increases degradation efficiency compared to glucose (Fig. S1). The ability of these isolates to degrade both types of aflatoxin in a linear association confirms that the use of AFG_2_ in our assays and screens is adequately representative of AFB_1_ degradation performance.

## Discussion

We investigated the possibility of using starch (instead of glucose) as the main carbon source in the growth medium to identify aflatoxin degraders from environmental samples. In this process, we identified new degrader species and found that starch in the environment resulted in an improved degradation phenotype for most isolates. Degradation levels varied in each isolate; however, generally, starch led to higher degradation levels compared to glucose. Additionally, the starch screen allowed for a more streamlined identification of AF degrader species, where growth on starch as the sole carbon source primed candidates for degradation of aflatoxin and facilitated the screening for better degraders.

Of importance in the data shown is how environmental carbon source can change the degradation profiles of certain species. We speculate that aflatoxin degradation is often carried out by off-target activity of carbon scavenging pathways. Given that pathways for utilizing more complex carbon sources are likely costly to microbial cells, it is expected that they are regulated to get expressed more when such complex carbon sources are present in the environment. The improvement to degradation by isolates when placed in a more complex carbon environment is aligned with such regulatory change and/or metabolic shift in microbes. This proposition justifies the use of a more complex carbon source, such as starch, to screen for AF degradation. By supplying the cells with a complex carbon as its sole carbon source, we are steering strains that can switch/adapt their metabolism toward complex carbons, and in the process, those that can break down AF. Further studies into the mechanisms behind this process are needed to control and improve this function for practical implementation.

Tested isolates were collected from areas that were not at predisposed risk for AF contamination and they likely did not have prior exposure to AF in the natural environment. The ability of these isolates to degrade AF indicates that the degradation capacity is not necessarily rare among bacterial species. Additionally, the taxonomic breakdown of the analyzed isolates shows a fairly diverse array of species that possess AF degradation ability, further indicating that AF degradation ability can arise in many species of bacteria.

To identify new species of AF degraders, our findings indicate using growth on starch medium is a good initial screening method due to its low cost, higher percentage of good degrader strains, and better outcome of strains with broader environmental working conditions. Downstream, utilizing a starch screen for samples that have a higher probability of pre-exposure to aflatoxins will be beneficial in finding new degrader strains that possess a high degradation ability. After identifying new strains with degradation potentials, follow-up steps are needed to ensure that the strain is suitable for practical applications. A necessary follow-up step is to examine the byproducts of degradation to ensure that the overall process is not toxic to animals/humans or the environment. Another important follow-up is to investigate the mechanisms of degradation, such as metabolic pathways, enzyme identification, and degradation by-product analysis to classify the diversity of possibilities and explore the next steps for improving the degradation performance.

## Methods

### Environmental isolates and culture mediums

Environmental samples were collected from in and around the Chestnut Hill area in Massachusetts (42.33547603, -71.16912210). These samples include soil, snow, leaf, tree trunk, doorknob, and phone screen swabs. Sterile DI water was added to the soil to create a suspension. All samples were struck out on standard LB agar and incubated for 1–3 days at room temperature and 28 °C. Individual colonies were then inoculated in either glucose or starch defined medium (medium screens performed from separate environmental samples) in a 96-well plate. Isolates were tested for growth via absorbance at OD_600_ on a BioTek Synergy Mx microplate reader.

Isolates were cultured in defined medium comprised of 1.5 g/L KH_2_PO_4_, 3.8 g/L K_2_HPO_4_·3H_2_O, 1.3 g/L (NH_4_)_2_SO_4_, 3.0 g/L sodium citrate·2H_2_O), 20.9 g/L MOPS, 1.1 mg/L FeSO_4_, 1 mL/L mixed vitamin solution (2 mg/L of biotin, 2 mg/L of folic acid, 10 mg/L of pyridoxine-HCl, 5 mg/L of thiamine-HCl·2H_2_O, 5 mg/L of riboflavin, 5 mg/L of nicotinic acid, 5 mg/L of D-Ca-pantothenate, 0.1 mg/L of vitamin B_12_, 5 mg/L of p-aminobenzoic acid, and 5 mg/L of lipoic acid), 1 mL/L SL-10 trace elements solution (10 mL/L of HCl (25%; 7.7 M), 1.5 g/L of FeCl_2_·4H_2_O, 70 mg/L of ZnCl_2_, 0.1 g/L of MnCl_2_·4H_2_O, 6 mg/L of H_3_BO_3_, 0.19 g/L of CoCl_2_·6H_2_O, 2 mg/L of CuCl_2_·2H_2_O, 24 mg/L of NiCl_2_·6H_2_O, and 36 mg/L of Na_2_MoO_4_·2H_2_O), 1 M MgCl_2_ (5 mL), 1 M CaCl_2_ (1 mL), and 10 mL/L mixed amino acid stock (1.6 g/L of alanine, 1 g/L of arginine, 0.4 g/L of asparagine, 2 g/L of aspartic acid, 0.05 g/L of cysteine, 6 g/L of glutamic acid, 0.12 g/L of glutamine, 0.8 g/L of glycine, 1 g/L of histidine monohydrochloride monohydrate, 2 g/L of isoleucine, 2.6 g/L of leucine, 2.4 g/L of lysine monohydrochloride, 0.6 g/L of methionine, 2 g/L of phenylalanine, 2 g/L of proline, 1 g/L of serine, 0.7 g/L of threonine, 0.3 g/L of tryptophan, 0.25 g/L of tyrosine, 2 g/L of valine, 2 g/L of adenine hemisulfate salt, and 2 g/L of uracil), with either glucose (4.0 g/L) or starch (4.0 g/L) as the carbon source. AFG_2_ and AFB_1_ (Cayman Chemical) was dissolved in LC-MS grade methanol to the final concentration of 1 mg/mL.

### Aflatoxin degradation assay

Cells or culture filtrates were aliquoted into sterile microcentrifuge tubes and aflatoxin was added according to desired final concentration (15–30 µg/mL) per well. Samples were arrayed in black glass-bottom 96-well plates (Nunc™ #165305 96-Well Optical Bottom) at a final volume of 150 µL per well. Standard controls of toxin alone (AFG_2_ in fresh medium) and no toxin (cells or filtrate alone) were used. A BioTek Synergy Mx multi-mode microplate reader was used to monitor optical density of cells at 600 nm and fluorescence of aflatoxin at an excitation of 380 nm and emission of 440 nm with a gain of 50. Reads were taken at 5 min intervals over 72 h (unless otherwise noted). Cultures usually started at an initial OD_600_ of 0.01 and were continuously shaking between reads. Typically, 2–3 replicates were used per condition. Sterile water was placed at the peripheral wells of the 96-well plate to contain evaporation.

### PCR and 16S rRNA gene sequencing

Isolates underwent colony PCR for 16S rRNA gene amplification. Cells were taken from agar plates, suspended in 10 µL Milli-Q, and lysed at 98°C for 15 min. Universal primers used for amplification and sequencing were: 27F (5’-AGAGTTTGATCCTGGCTCAG-3’) and 1492R (5’-GGTTACCTTGTTACGACTT-3’). PCR product was sent for Sanger sequencing, both forward and reverse (from each end of the PCR product), at Eurofins Genomics. Resulting forward and reverse sequences were merged to obtain the entire 16S rRNA gene sequence. We then used BLASTn (NCBI) to find the closest sequence match in the Core Nucleotide Database (core nt) for strain identification.

### Phylogenetic tree

Based on BLASTn analysis and the closest match for each isolate, we used the GenBank reference sequences for the creation of the phylogenetic tree through multi-sequence alignment. From the original 16S rRNA gene sequences, we first created the alignment file using CLUSTALW^37^. The resulting .aln file was entered into IQ-TREE^38^ to generate the consensus phylogenetic tree and the bootstrap confidence values^39,40^. In the bootstrap algorithm, 1000 permutations were used. The tree file was visualized and annotated using Interactive Tree of Life (iTOL, version 7.0)^41^.

### Data analysis

Raw data from the aflatoxin degradation assay was processed using Matlab generated codes to measure growth and degradation characteristics. Background fluorescence (no toxin control) is subtracted from the readings to remove fluorescence from sources other than the toxin. The readouts are also normalized to fluorescence data from no cell controls to remove the effect of fluorescence loss due to bleaching or other causes over time with an additional normalization to account for fluorescence loss due to cell scattering (Fig. S4). To convert the fluorescence readout to the corresponding toxin concentration, we employ a calibration curve based on measurements of a set of known toxin concentrations^24^. After normalization, degradation efficiency is calculated as the percentage of toxin removed during the testing period (48 h).

### Statistical analysis

To compare the odds ratio between good degraders and poor or non-degraders obtained from the starch versus glucose isolation (Fig. 1), we performed Fisher’s exact test using Matlab software (version R2021a, MathWorks Inc., Natick, MA, USA) through the function fishertest. To compare the growth rates, carrying capacities, and AFG_2_ degradation efficiency between starch- versus glucose-isolates (Fig. 3), we used Mann-Whitney U test using the Matlab function ranksum. To test the significance of binomial outcomes, we used the function myBinomTest (written by Matthew Nelson^42^) in Matlab, assuming a two-sided distribution and comparing the observed outcome with an unbiased probability of 0.5.

## Electronic supplementary material

Below is the link to the electronic supplementary material.

Supplementary Material 1

## Data Availability

Raw data and codes used for analysis of the data in this study are shared on GitHub at https://github.com/nsandlin7/EI_starch. Merged 16S rRNA gene sequences and corresponding sequences from closest matches in the GenBank database are available in the same GitHub repository.
