# Supplementary material for: Starch can expedite the screening for bacterial aflatoxin degraders
Source: Sci Rep. 2024 Dec 30;14:31961. doi: 10.1038/s41598-024-83511-3 (PMC11685649; doi:10.1038/s41598-024-83511-3)
Supplement: Supplementary file 1 — Supplementary Material 1 [file 41598_2024_83511_MOESM1_ESM.pdf]

Supplemental figures

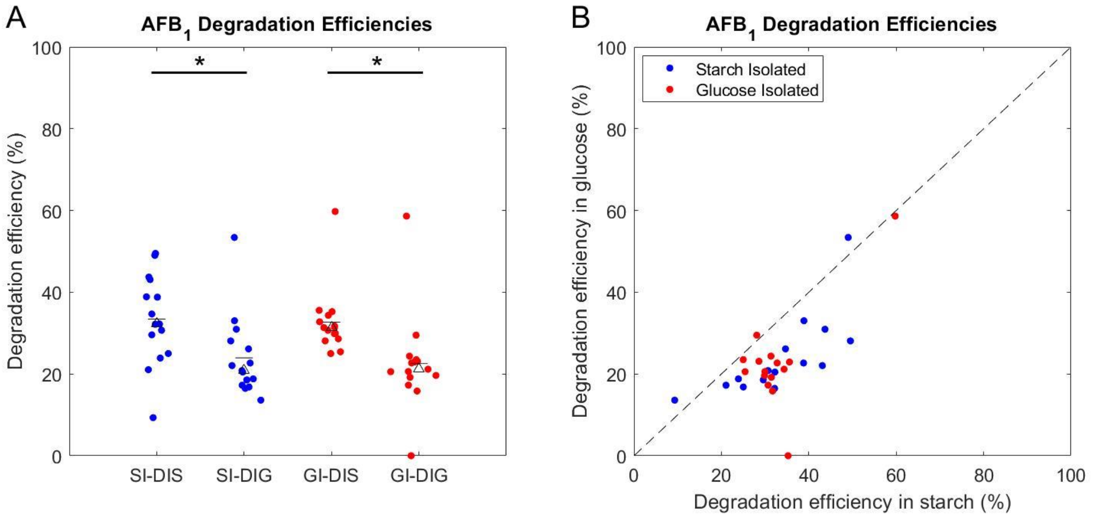

Figure S1. AFB_1 degradation is improved when tested in starch medium. Isolates were tested for their AF degradation efficiency when grown in starch and glucose defined media. Starch isolated strains are shown in blue and glucose isolated strains are shown in red. A) Degradation efficiency, shown as percent AF degraded in 48 hours, grouped by isolation and testing medium. Testing in starch medium is indicated by DIS and testing in glucose medium is indicated by DIG. Degradation efficiency is shown as percent AF degraded in 48 hours. The marked triangles indicate the group's median, while the marked dash indicates the group's mean. B) Degradation efficiencies for each isolate in both media. The dotted line represents the same efficiency between the two media. Each dot is the mean of 2 replicates per culturing condition. * = $p<0.05$, Mann-Whitney U test.

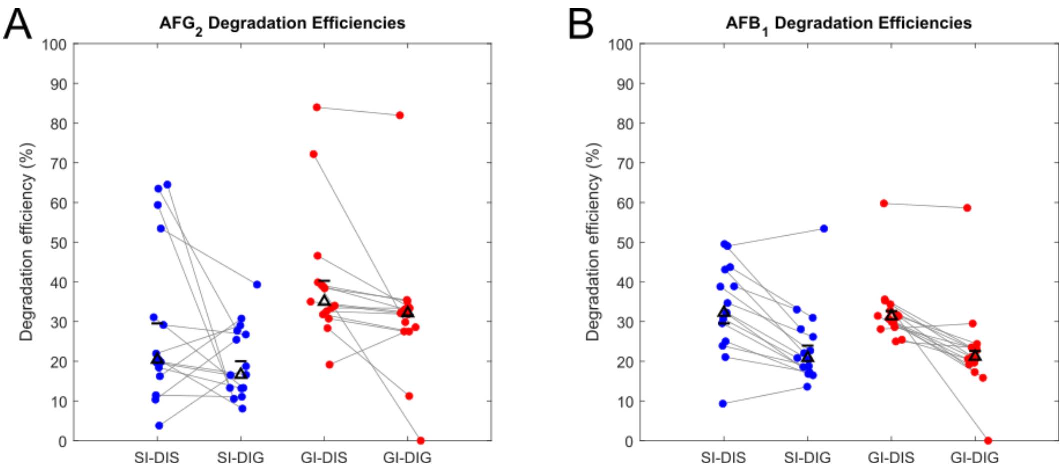

Figure S2. A majority of isolates show better degradation in the starch medium, compared to the glucose medium. Isolates were tested for their AF degradation efficiency when grown in starch and glucose defined media. Starch isolated strains are shown in blue and glucose isolated strains are shown in red. A) AFG_2 degradation efficiency, shown as percent AFG_2 degraded in 48 hours, decreased for 11 out of 15 ($p = 0.12$, not significantly different from random) among glucose-isolated strains and for 14 out of 15 ($p = 0.001$, significantly different from random) among starch-isolated strains. B) AFB_1 degradation efficiency, shown as percent AFB_1 degraded in 48 hours, decreased for 13 out of 15 ($p = 0.007$, significantly different from random) among starch-isolated strains and for 14 out of 15 ($p = 0.001$, significantly different from random) among glucose-isolated strains. All p-values here are from testing whether the observed events in a binomial distribution are significantly different from 0.5.

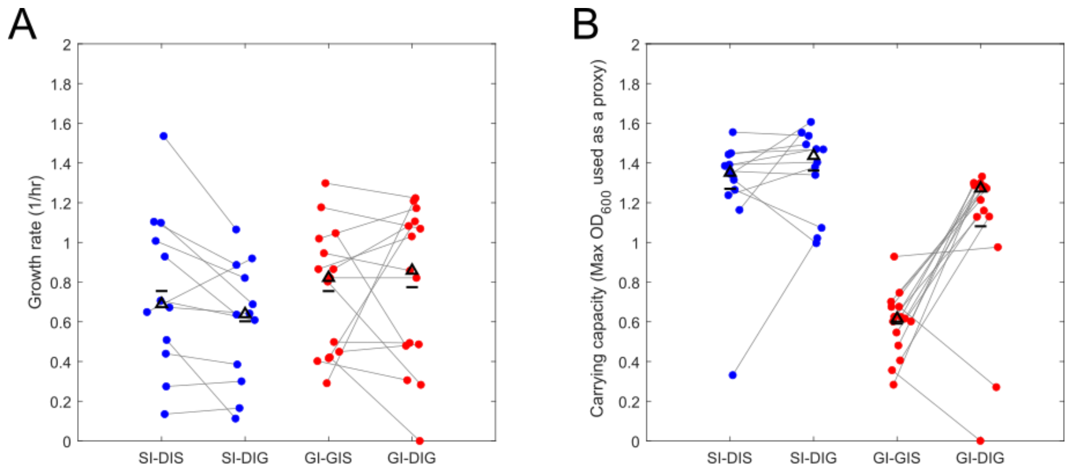

Figure S3. A majority of isolates grow to a higher carrying capacity in the glucose medium, compared to the starch medium. (A) The growth rate and (B) the carrying capacity (using OD_{600} as a proxy for cell density) of starch-isolated (blue) and glucose-isolated (red) strains were quantified. (A) Among starch-isolated strains, a majority of strains (9 out of 12; p = 0.146, not significantly different from random) grew faster in starch medium compared to glucose medium. Among glucose-isolated strains, there was no clear pattern of faster growth in starch versus glucose media (7 out of 13 grew faster in glucose medium, whereas 6 out of 13 grew faster in starch medium; p = 1, not significantly different from random). (B) Among starch-isolated strains, there was no clear pattern of higher carrying capacity in starch versus glucose media (6 out of 12 showed higher carrying capacity in starch medium, whereas the other 6 out of 12 showed higher carrying capacity in glucose medium; p = 1, not significantly different from random). Among glucose-isolated strains, a majority of strains (11 out of 13; p = 0.022, significantly different from random) showed higher carrying capacity in glucose medium compared to starch medium. All p-values here are from testing whether the observed events in a binomial distribution are significantly different from 0.5.

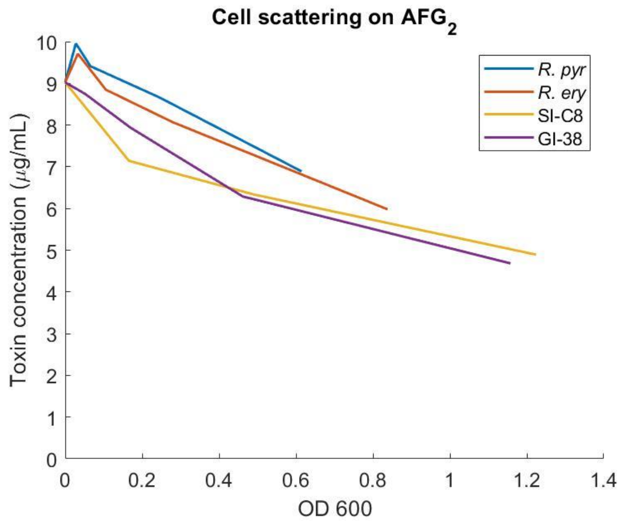

Figure S4. Cell-scattering influences the fluorescence readout of AF. The same concentration of AFG_2 was added to serial dilutions of cells immediately prior to measuring fluorescence in our FL assay. Readings reflect the effects of fluorescence scattering due to cell density. Linear slope for each representative strain was calculated and averaged to set as the normalization of cell scattering during growth in our FL assay.
